# The passing of an AIDS Activist: in memorium, Archbishop Desmond Mpilo Tutu

**DOI:** 10.1002/jia2.25878

**Published:** 2022-01-20

**Authors:** Chris Beyrer, Linda‐Gail Bekker

**Affiliations:** ^1^ Desmond M. Tutu Professor in Health and Human Rights, Department of Epidemiology Johns Hopkins Bloomberg School of Public Health Baltimore Maryland USA; ^2^ Faculty of Health Sciences Institute of Infectious Disease and Molecular Medicine Desmond Tutu HIV Center Cape Town South Africa

His Grace, Archbishop Emeritus of Cape Town, Desmond M. Tutu, was a man known most for his unforgettable leadership in the struggle against the Apartheid regime in his beloved South Africa. Archbishop Tutu imagined the Rainbow Nation to come, and helped lead his oppressed, often grieving, and sometimes deeply (and justifiably) enraged people to envision a brighter, more tolerant future. He was that rarest of leaders – a man who had vision and compassion coupled with toughness, a savvy political mind and an unwavering moral compass. He embodied a radical inclusivity that tolerated no one being excluded from compassion and care. And when he said that no one was excluded from his Father's house – he meant no one. In the early years of the AIDS pandemic when so many were being excluded, shunned and shamed, his inclusivity was truly transformative and empowering for millions.

As Archbishop Tutu's Rainbow Nation struggled to emerge, and Nelson Mandela was freed from decades of incarceration to lead the establishment of a new democracy, Tutu graciously stepped aside and let the statesmen lead. But he held true to his principles and to giving voice to the voiceless. When a corrupt faction of the African National Congress came to power, and was stealing his people's future, he was as ferocious a critic of their misrule as he had been of Apartheid. He never lost sight of the people who needed him most – including the millions of urban and rural poor living in the Townships ringing South Africa's cities, and those at the margins – LGBTQ people and people living with HIV/AIDS and tuberculosis.

With his unerring sense of where the right and just side of any issue lay, Tutu stood up to Thabo Mbeki's deadly anti‐scientific AIDS denialism. He joined the activists on the streets pressing for affordable antiviral drugs for Africa and the world. And he lent his name and his blessings to countless vitally important HIV causes, charities and programs. Having himself faced and overcome polio as a child, potentially crippling spinal tuberculosis as an adolescent and facing prostatic carcinoma in his latter years, he had a remarkable empathy and compassion for the individuals both directly and indirectly affected by HIV and tuberculosis. He enthusiastically served as patron to the Desmond Tutu HIV Centre and Foundation, also lending his name and support to the Desmond Tutu Tuberculosis Centre, and was a keen supporter and patron of the Tygerberg Childrens’ Hospital Trust and the Philani Maternal, Child Health and Nutrition Trust – all of which have actively sought to reduce suffering among the poorest communities hardest hit by HIV and tuberculosis. We will be forever grateful for his hands‐on guidance, interest, wisdom, enduring endorsement and encouragement.

A man of the deepest faith, Tutu vigorously embraced the science of HIV prevention, treatment and care. In 2016, the International AIDS Conference was due to return to Africa for the first time since the landmark AIDS 2000 Conference, and it was again coming to Durban. The IAS – International AIDS Society – leadership could think of no better person to open the Conference, and to fill the extraordinary role played by President Mandela when he had spoken at AIDS 2000, saying simply, “HIV is the cause of AIDS.” Archbishop Tutu was already losing strength and fighting several health challenges by that time, but he agreed, with the caveat that should he not be able to attend in person, he would provide a pre‐taped video address. Unfortunately, he was unable to be with the HIV community in person in Durban that July. Still, his videotaped message was compellingly clear — we still had not done enough to combat stigma, to protect adolescents and young women from HIV, and we still had a long way to go towards achieving universal access to healthcare. His blessing too was wonderfully poignant, as he thanked and blessed all the healthcare workers and extended his love to all living with virus and not. He had another message for us all in that speech — and it was not on HIV or TB but on climate change. He made clear that he considered it a great moral injustice that those who had done the least to alter the climate — the world's poor — were already paying the greatest price.

The spiritual core of Archbishop Tutu's faith, and of his AIDS activism and advocacy, was the African concept of *Ubuntu. Ubuntu* speaks to our shared mutual interdependence. It recognizes the principle that one's own humanity, one's own dignity, depends on the humanity and dignity of others. If one of our human family, or one group, like people living with HIV, are denied their full humanity, then so are we all. The imperative of *Ubuntu*, and of the Archbishop's lifelong ministry, was to extend the circle of our love and concern to all our brothers and sisters. He did not simply preach this. He lived it. And in fighting Apartheid, which by diminishing the human dignity of Black South Africans diminished the dignity of all who participated in it and all who benefitted from it, he made real the tenets of his belief.

Humanity has lost a great soul. The HIV/AIDS movement has lost an irreplaceable ally. Yet, the passing of this remarkable man is not simply a cause of sadness for those who loved him. His teachings and his legacy will live and last, because he was always on the side of the oppressed and he fought injustice with a mighty heart. Truly ending AIDS will require not only scientific and programmatic advances, but also *Ubuntu*. We must extend our love and concern to all — no exceptions. He taught us that. And he can leave no greater legacy than the charge to us all to do better. Our shared humanity depends on it.

Chris Beyrer

IAS Past President (2014–2016)

Linda‐Gail Bekker

IAS Past President (2016–2018)

## FUNDING

None declared.

## COMPETING INTERESTS

The authors declare no competing interests.

## AUTHORS’ CONTRIBUTIONS

CB and LGB have contributed to the preparation of the manuscript, read and approved the final draft.

